# Saliva microbiome changes in thyroid cancer and thyroid nodules patients

**DOI:** 10.3389/fcimb.2022.989188

**Published:** 2022-08-11

**Authors:** Junjun Jiao, Youli Zheng, Qingyu Zhang, Degeng Xia, Li Zhang, Ning Ma

**Affiliations:** ^1^ Hospital of Stomatology, Jilin University, Changchun, China; ^2^ The School and Hospital of Stomatology, Tianjin Medical University, Tianjin, China

**Keywords:** thyroid cancer, thyroid nodules, microbiota, 16S rRNA sequencing, oral, clinical index

## Abstract

**Objective:**

Thyroid disease has been reported to associate with gut microbiota, but the effects of thyroid cancer and thyroid nodules on the oral microbiota are still largely unknown. This study aimed to identify the variation in salivary microbiota and their potential association with thyroid cancer and thyroid nodules.

**Methods:**

We used 16S rRNA high-throughput sequencing to examine the salivary microbiota of thyroid cancer patients (n = 14), thyroid nodules patients (n = 9), and healthy controls (n = 15).

**Results:**

The alpha-diversity indices Chao1 and ACE were found to be relatively higher in patients with thyroid cancer and thyroid nodules compared to healthy controls. The beta diversity in both the thyroid cancer and thyroid nodules groups was divergent from the healthy control group. The genera Alloprevotella, Anaeroglobus, Acinetobacter, unclassified Bacteroidales, and unclassified Cyanobacteriales were significantly enriched in the thyroid cancer group compared with the healthy control group. In contrast, the microbiome of the healthy controls was mainly composed of the genera Haemophilus, Lautropia, Allorhizobium Neorhizobium Pararhizobium Rhizobium, Escherichia Shigella, and unclassified Rhodobacteraceae. The thyroid nodules group was dominated by genre uncultured Candidatus Saccharibacteria bacterium, unclassified Clostridiales bacterium feline oral taxon 148, Treponema, unclassified Prevotellaceae, Mobiluncus, and Acholeplasma. In contrast, the genera unclassified Rhodobacteraceae and Aggregatibacter dominated the healthy control group. The study also found that clinical indicators were correlated with the saliva microbiome.

**Conclusion:**

The salivary microbiota variation may be connected with thyroid cancer and thyroid nodules.

## Introduction

Thyroid nodules are common in clinical practice, with approximately 60% of adults harboring one or more thyroid nodules, and the majority of them are benign. Palpable thyroid nodules occur in about 4-7% of the population, but only about 8% to 16% of thyroid nodules harbor thyroid cancer (Burman and Wartofsky, 2015). Thyroid cancer is the most common endocrine malignancy and is responsible for 586000 cancer cases worldwide, with a higher global incidence rate in women (Ferlay et al., 2015; Sung et al., 2021). Over the past 3 decades, the global incidence rate of thyroid cancer has rapidly increased by 300%, but the mortality rate remains much lower and comparatively stable (Kitahara and Sosa, 2016; Lortet-Tieulent et al., 2019; Sung et al., 2021). As a result, though better-prognosis thyroid cancer may seem like a “good cancer,” previous studies have shown that thyroid cancer survivors with a better prognosis have a poorer quality of life than normal people and other worse-prognosis cancer survivors (Singer et al., 2012; Goswami et al., 2018; Chan et al., 2021). Some risk factors, such as familial influences, sex, obesity, hormonal exposure, smoking, and environmental risk factors, may increase the risk of thyroid cancer, but the etiology of thyroid cancer remains poorly understood to date (Kim et al., 2020). Interestingly, some studies have suggested that thyroid cancer and thyroid nodules may have a relationship with microbiota, and the risk factors of thyroid diseases such as hormones and obesity are also linked to the composition and diversity of microbiota, implicating the microbiota perhaps play a role in thyroid cancer and thyroid nodules (Maruvada et al., 2017; Stefura et al., 2021; Dong et al., 2021).

It is known that the relationship between oral microbiota and cancer has been extensively studied. Several oral taxa have been shown to promote cancer development through different mechanisms such as inhibiting apoptosis, activating cell proliferation, promoting cell invasion, inducing chronic inflammation, and directly producing carcinogens (Irfan et al., 2020; Tuominen and Rautava, 2021). Indeed, a growing number of studies have shown associations between changes in the oral microbiota and a wide variety of cancer types such as colorectal cancer, esophageal cancer, throat cancer, liver cancer, and lung cancer (Peters et al., 2017; Yang et al., 2018; Wang et al., 2019; Li et al., 2020; Uchino et al., 2021). These studies suggest that the oral microbiota may provide a potential biomarker for some cancer diagnoses. Additionally, a study reported that salivary microbial profiles change with increasing serum TSH (thyroid-stimulating hormone), which is reflected in increased taxa diversity, changes in community structure, and species composition (Dong et al., 2021). And there is evidence that serum TSH at presentation is an independent predictor of differentiated thyroid cancer (DTC) and the thyroid nodules are at increased risk for malignancy with elevated serum TSH concentrations within the normal range (Haymart et al., 2008). Another study suggests that sex hormones affect the change of oral microbiota, while estrogen has been proven to play an essential role in thyroid nodules (Kim et al., 2010; Lin et al., 2018). Moreover, recent evidence has revealed that thyroid cancer and thyroids nodules are associated with gut microbiota (Feng et al., 2019; Zhang et al., 2019; Li et al., 2021). Oral-to-gut and gut-to-oral microbial transmission can modulate the pathogenesis of various human diseases, suggesting the potential interaction between thyroid diseases and oral microbiota despite the paucity of studies on the role of the oral microbiota in thyroid cancer (Kitamoto et al., 2020; Park et al., 2021).

Therefore, we compared the microbiota in human saliva from healthy controls and patients with thyroid cancer and thyroid nodules using 16s rRNA gene sequencing to identify the potential relationship between the oral microbiota and thyroid cancer and thyroid nodules.

## Materials and method

### Participant recruitment

This study enrolled patients with thyroid cancer and thyroid nodules from March 2022 to May 2022 in North China and recruited healthy people as controls from the resident community in Qingdao, China. The inclusion criteria for patients with thyroid disease were as follows: (1) clinically diagnosed with thyroid nodules by ultrasound; (2) Some high-grade thyroid nodules such as TI-RADS level 4a and thyroid cancer identified by pathological examination. The healthy controls were frequently matched with the thyroid cancer and thyroid nodules patients for age, gender, and body mass index (BMI); no healthy controls had thyroid lesions. The following exclusion criteria were applied to all groups: pregnancy; lactation; cigarette smoking; alcohol addiction; hypertension; diabetes mellitus; kidney disease; BMI < 18.5; BMI > 30.3; recent (< 3 months prior) use of antibiotics, probiotics, prebiotics, symbiotics, hormonal medication; known history of disease with an autoimmune component; and history of malignancy; unwilling to sign the informed consent.

This study followed the Declaration of Helsinki on medical protocols and ethics and obtained approval from the Regional Ethical Review Board of the Hospital of stomatology, Jilin University. All participants have been informed of the intention of the sample collection and signed written informed consent.

### Anthropometric, biochemical measurements, and group definition

Demographic information, including height, gender, and body mass index (BMI), was obtained during subject recruitment. The clinical indicators were obtained from the participants who were required to fast for 8 hours. Two independent ultrasonography clinicians verified the description of the thyroid nodules and classified them according to the Kwak-TIRADS criteria (Kwak et al., 2011). At least two pathologists confirmed the diagnosis of thyroid cancer.

We recruited 14 thyroid cancer patients, 9 thyroid nodules patients, and 15 healthy controls. All the thyroid cancer patients we recruited were papillary thyroid cancer (PTC). All the thyroid nodules patients were not malignant. All participants live in the northern coastal provinces of CHINA, where the typical diet includes steamed bread, meat, seafood, vegetables, and fruits.

### Sample collection

Saliva samples were obtained from recruited patients before brushing their teeth and eating breakfast. Subjects started by rinsing the mouth with stroke-physiological saline solution before sampling. Then, 2 to 5 ml saliva was collected in a 10ml sterile Eppendorf tube, transferred to the laboratory immediately in an ice box, and stored at -80℃ until sequencing.

### DNA extraction, amplification, and high-throughput sequences

The genomic DNA was extracted from saliva samples with a QIAamp DNA Mini Kit (Qiagen, Valencia, CA, USA) according to the manufacturer’s instruction and then quantified using a spectrophotometer and 1% agarose gel electrophoresis.

The V3-V4 variable regions were high-throughput sequenced by Biomarker Technologies Co, Ltd. (Beijing, China) using an Illumina NovaSeq6000 platform according to established protocol. A sample-unique 8-base barcode was contained in each forward primer. The V3-V4 region of the bacterial 16S rRNA gene was amplified using the universal forward primer 338F: 5′- ACTCCTACGGGAGGCAGCA-3′ and reverse primer 806R: 5′-GGACTACHVGGGTWTCTAAT-3′. The PCR program was as follows: 95°C, 5 min; 25 cycles of 30 s at 95°C, 30 s at 50°C, and 40 s at 72°C; and a final extension of 72°C for 7 min. PCRs were performed with a 10μL reaction mixture containing 5 μL of KOD FX Neo Buffer, 2 μL of 2mMDNTPS, 0.3 μL of each primer (10μm), 0.2 U of KOD FX Neo, and 15 ng of template DNA.

1.8% agarose gel electrophoresis separated the PCR products. Purified amplicons from different samples were pooled in equimolar and sequenced in the Illumina NovaSeq6000 platform.

### Data processing

Merging the paired-end reads used FLASH v1.2.7 (Yang et al., 2020). According to the default settings, the minimum overlap length and maximum mismatch ratio of merged paired-end reads were 10bp and 0.2, respectively. Simultaneously, the tags with more than six mismatches were eliminated. Quality control was performed using Trimmomatic (version 0.33) to determine the merged tags with an average quality score < 20 in a 50bp sliding window and remove those shorter than 350bps (Sheng et al., 2019). Denoising and removing the chimeras in dada2 *via* QIME2 2020.6 and obtaining amplicon sequence variations (ASVs) feature sequences (Callahan et al., 2016; Bolyen et al., 2019).

### Statistical analysis

Data analyses were undertaken using SPSS (version 25.0) and R software (version 4.20). P values < 0.05 were considered statistically significant. For continuous variables with normal distributions and equal variance, including BMI (body mass index), WBC (white blood cell), RBC (red blood cell), PLT (platelet), TP (total protein), and GLU (glucose), analysis of variance (ANOVA) was carried out for group comparisons. Continuous variables conforming to normal distribution but with unequal variance included AST (aspartate transaminase) and FT3 (free triiodothyronine), which were analyzed by the ANOVA after Welch correction. Wilcoxon rank-sum test was utilized to detect the continuous variable without normality, including age, ALT (alanine aminotransferase), TBIL (total bilirubin), TG (triglyceride), CHOL (total cholesterol), HDL (high-density lipoprotein), LDL (low-density lipoprotein), FT4 (free thyroxine), TSH (thyroid-stimulating hormone), TGAb (antithyroglobulin antibody), TPOAb (antithyroperoxidase antibody), and PTH (parathyroid hormone). The Fisher exact test was assessed for the data of categorical variables of gender.

**Figure 1:**
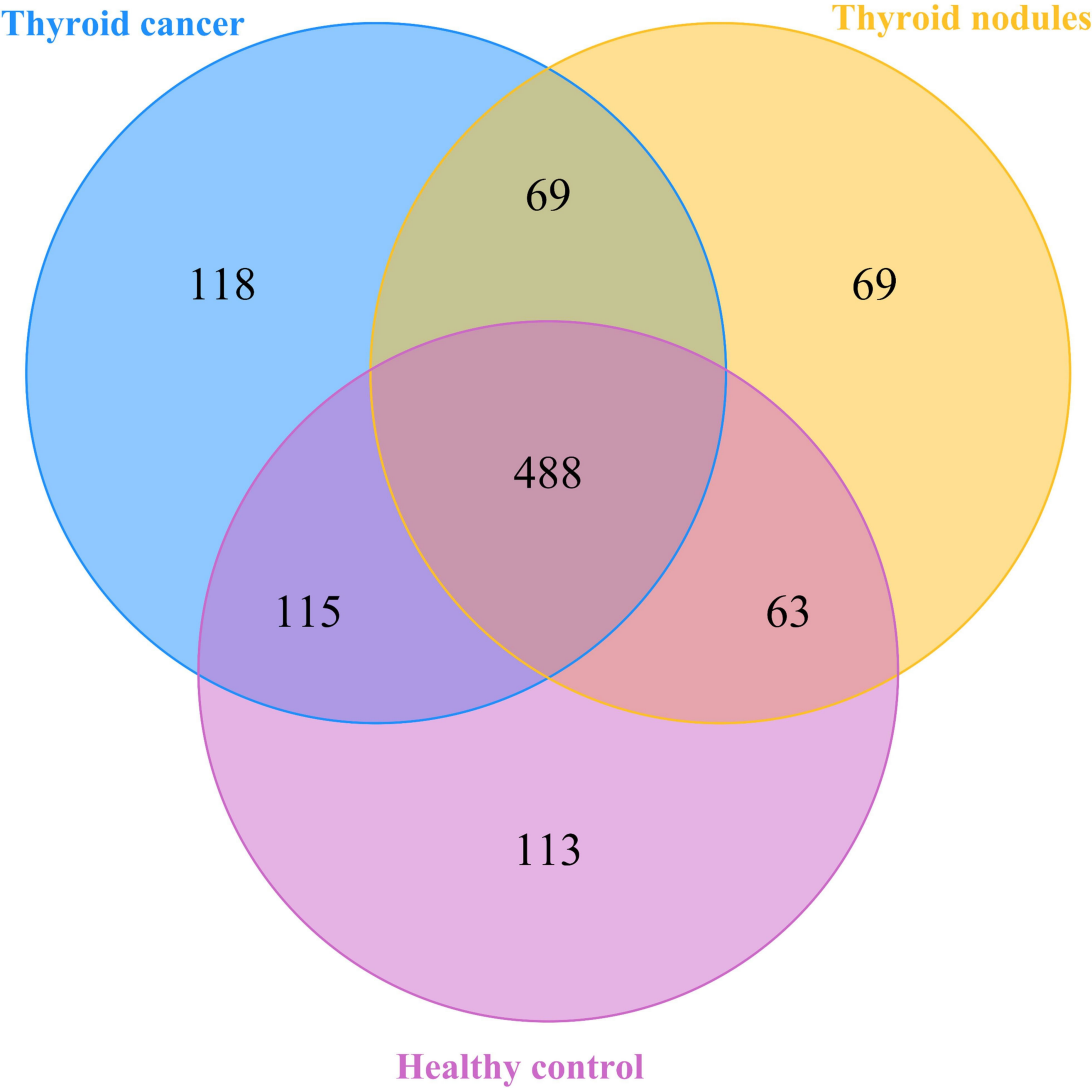
A Veen diagram comparing the distribution of amplicon sequence variations (ASVs) among the thyroid cancer, thyroid nodules, and healthy control groups. The different colors represent different groups. The overlap represents shared bacteria.

**Figure 2:**
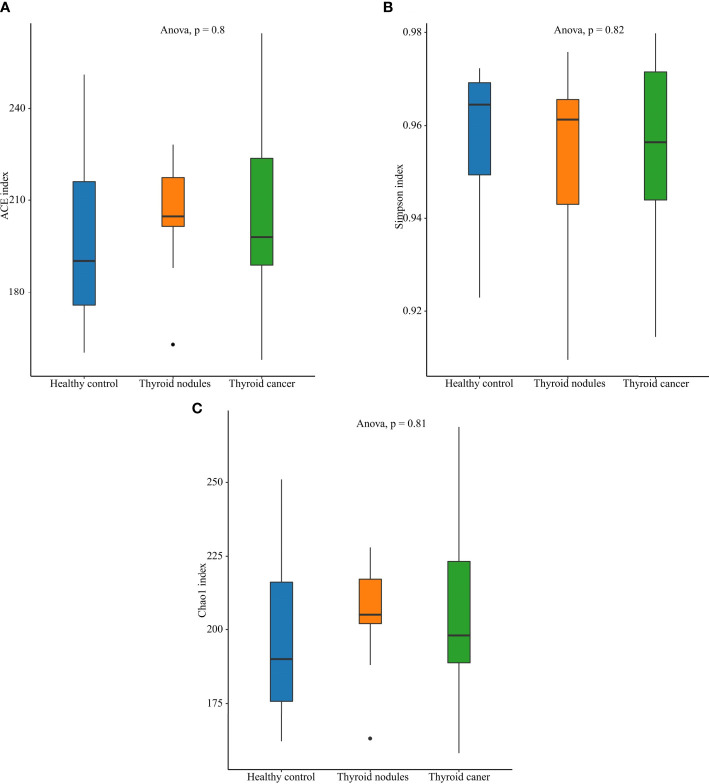
Alpha diversity of the saliva microbiome in thyroid cancer patients, thyroid nodules patients, and healthy controls. **(A)** Richness of saliva microbiota assessed by ACE. **(B)** Simpson is a diversity index. **(C)** Richness of saliva microbiota assessed by Chao1.

We drew a Venn diagram to depict the unique or shared ASVs in three groups (**Figure 1**) (Chen and Boutros, 2011). Alpha diversity was calculated on the basis of the gene profile for each sample, based on the Simpson, Chao1, and ACE index. Alpha-diversity estimates were computed using QIIME2 2020.6 software (**Figure 2**) (Bolyen et al., 2019). The ANOVA method was used to examine the differences between the three groups.

The β-diversity index comparisons were used to compare differences in microbial diversity among the three groups using principal coordinate analysis (PCoA) based on the unweighted UniFrac analysis. The weighted UniFrac used to calculate the Phylogenetic distances of the system took the evolutionary distance between the species into account to compare the microbial diversity differences (Lozupone and Knight, 2005). Permutational multivariate analysis of variance (PERMANOVA) was used to test the significance of the differences among the three groups.

LEfSe (Linear discriminant analysis Effect Size) can be used to analyze the differences between groups and look for biomarkers with statistical differences between groups.

First, Kruskal-Wallis (KW) was used to detect features with significant differences in abundance between different groups, and significance was set to 0.05; Subsequently, a set of pairwise tests among subclasses was performed to use the Wilcoxon rank-sum test, with significance set at 0.5; At last, linear discriminant analysis (LDA) was used to downscale the data and assess the influence of species with significant differences. Lefse was performed to identify the most discriminating taxa in groups from phylum to genus, and taxa with an LDA score > 2.0 were considered the most discriminating species (Segata et al., 2011). Correlations between the clinical parameters and different microbiota were quantitatively evaluated using the Pearson correlation coefficient, and the correlation was presented using a heatmap.

## Result

### Study population

To establish the salivary microbiota characteristics of patients with thyroid cancer and thyroid nodules, we analyzed 38 saliva samples from 38 participants (14 patients with thyroid cancer, 9 patients with thyroid nodules, and 15 healthy controls) using 16S rRNA gene sequencing. No significant differences were noticed in the clinical parameters (p > 0.05) among the three groups. The demographic and biochemical variables of patients with thyroid cancer and thyroid nodules were summarized in **Table 1**.

**Table 1:** Basic characteristics of participants.

Variables	Thyroid cancer	Thyrois nodules	Healthy control	P- value
Gender (M: F)	11:3	9:0	12:3	0.40
Age (years)	54.50 (41.30, 58.80)	54.00 (49.00, 57.00)	55.00 (36.50, 59.50)	0.91
BMI (kg/m^2^)	24.20 ± 3.380	23.70 ± 2.88	23.20 ± 2.65	0.71
WBC (10^9^/L)	5.52 ± 1.10	5.84 ± 1.31	5.63 ± 1.16	0.82
RBC (10^12^/L)	4.59 ± 0.33	4.48 ± 0.39	4.48 ± 0.47	0.72
PLT (10^9^/L)	239.71 ± 58.01	221.44 ± 66.97	226.00 ± 40.89	0.69
ALT (U/L)	18.00 (13.30, 26.80)	17.00 (15.00, 18.00)	18.00 (17.00, 21.50)	0.55
AST (U/L)	22.00 ± 6.88	18.89 ± 1.83	17.13 ± 6.40	0.19
TP (g/L)	71.11 ± 6.42	71.22 ± 7.41	70.67 ± 3.92	0.95
TBIL (umol/L)	10.60 (9.44, 14.40)	12.00 (8.57, 14.70)	11.00 (9.00, 12.50)	0.88
GLU (mmol/L)	5.15 ± 0.57	5.42 ± 0.66	5.19 ± 0.48	0.55
TG (mmol/L)	1.03 (0.74, 0.81)	1.10 (1.01, 1.21)	0.98 (0.81, 1.20)	0.25
CHOL (mmol/L)	4.38 (4.08, 4.78)	4.39 (4.35, 4.77)	4.29 (4.04, 4.52)	0.14
HDL (mmol/L)	1.31 (1.20, 1.38)	1.04 (0.97, 1.49)	1.32 (1.21, 1.38)	0.40
LDL (mmol/L)	2.96 (2.80, 3.47)	2.78 (2.72, 3.36)	2.91 (2.80, 3.21)	0.59
FT3 (Pmol/L)	5.14 ± 1.48	4.90 ± 0.44	NA	0.58
FT4 (Pmol/L)	15.20 (14.00, 19.40)	17.10 (15.20, 18.80)	NA	0.55
TSH (uIU/ml)	1.940 (1.43, 4.35)	2.27 (1.17, 2.39)	NA	0.61
TG-Ab (IU/ml)	16.50 (15.00, 17.80)	16.40 (14.60, 20.10)	NA	0.90
TPO-Ab (IU/ml)	9.50 (9.00, 10.20)	10.00 (9.00, 10.30)	NA	0.41
PTH (pg/ml)	37.30 (29.70, 47.70)	38.90 (36.00, 41.70)	NA	0.66
				

The analysis of variance (ANOVA) was carried out to compare BMI, WBC, RBC, PLT, TP, and GLU. The ANOVA analyzed AST and FT3 after Welch correction. Wilcoxon rank-sum test was used to detect age, ALT, TBIL, TG, CHOL, HDL, LDL, FT4, TSH, TGAb, TPOAb, and PTH. The Fisher exact test was assessed for the data of categorical variables of gender.

### Bacterial composition in saliva in patients with thyroid cancer and patients with thyroid nodules and HCs

After filtering poor-quality reads, we collected an average of 79,817 clean reads per sample and 1035 ASVs in total. Among the 1035 ASVs found in the 38 samples, 488 were shared among all three groups. We identified 790 ASVs, 689 ASVs, and 779 ASVs in the thyroid cancer, thyroid nodules, and healthy control groups, respectively (**Figure 1**). Additionally, 118 ASVs were found only in thyroid cancer group, 69 were observed only in the thyroid nodules group, and 113 unique were observed in the healthy control group. Sixty-nine ASVs were shared by patients with thyroid cancer and those with thyroid nodules, 63 were found in the thyroid nodules group and healthy control group, and 115 were observed in patients with thyroid cancer and healthy controls.

### Alpha-diversity in three groups

An increase in abundance (CHAO1, ACE) was observed in the thyroid cancer group and thyroid nodules group compared with the normal control group, though the differences showed little statistical significance among the three groups (ANOVA, p > 0.05). Also, the Shannon index, which reflects community abundance and evenness, did not show significant differences among the three groups (ANOVA, p > 0.05). Hence, there was no significant difference in the alpha diversity among the three groups (**Figure 2**).

### Salivary microbiota composition and abundance in thyroid cancer and thyroid nodules patients, and healthy controls

**Figure 3:**
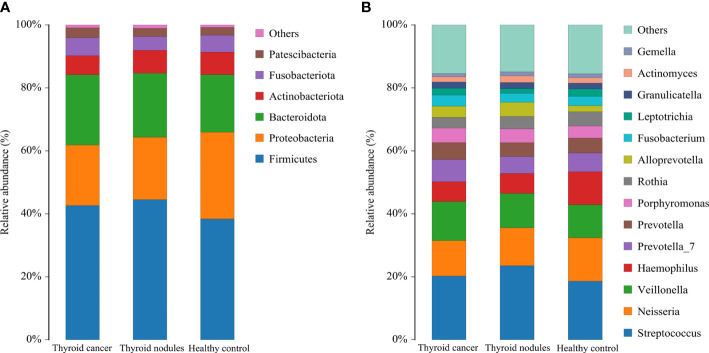
Relative abundance of dominant species in patients with thyroid cancer, patients with thyroid nodules, and healthy controls. **(A, B)** shows the relative abundance of the major phylum and genus, respectively. Each column of the bar graph represents a group, and each patch represents the proportion of a class of microbes in that group.

The dominant abundance of bacterial species at the genus and phylum levels was shown in **Figure 3**. The dominant phyla among the thyroid cancer, thyroid nodules, and healthy control group were Firmicutes, Proteobacteria, Bacteroidata, Actinobacteriota, and Fusobacteriota. We found that the above bacteria ranked differently in various groups. The Firmicutes accounted for 42.63%, 44.53%, and 36.38% in thyroid cancer, thyroid nodules, and healthy control group, respectively. The proteobacteria accounted for 19.21%, 19.80% and 27.52% in three groups respectively, the Bacteroidata accounted for 22.35%, 20.34%, and 18.31%, the Actinobacteriota accounted for 5.99%, 7.29%, and 7.12%, and the Fusobacteriota accounted for 5.70%, 4.39%, and 5.42%. In addition, the most prevalent genera were Streptococcus, Neisseria, Veillonella, Haemophilus, Prevotella 7, Prevotella, and Porphyromonas.

In conclusion, Firmicutes was the most prevalent phylum in all three groups. However, Proteobacteria only ranked second in the healthy control group, and Bacteroidata was more abundant in the thyroid cancer and thyroid nodules group than in the healthy control group.

### Variation in the composition and diversity of community of thyroid cancer, thyroid nodules, and healthy control group

**Figure 4:**
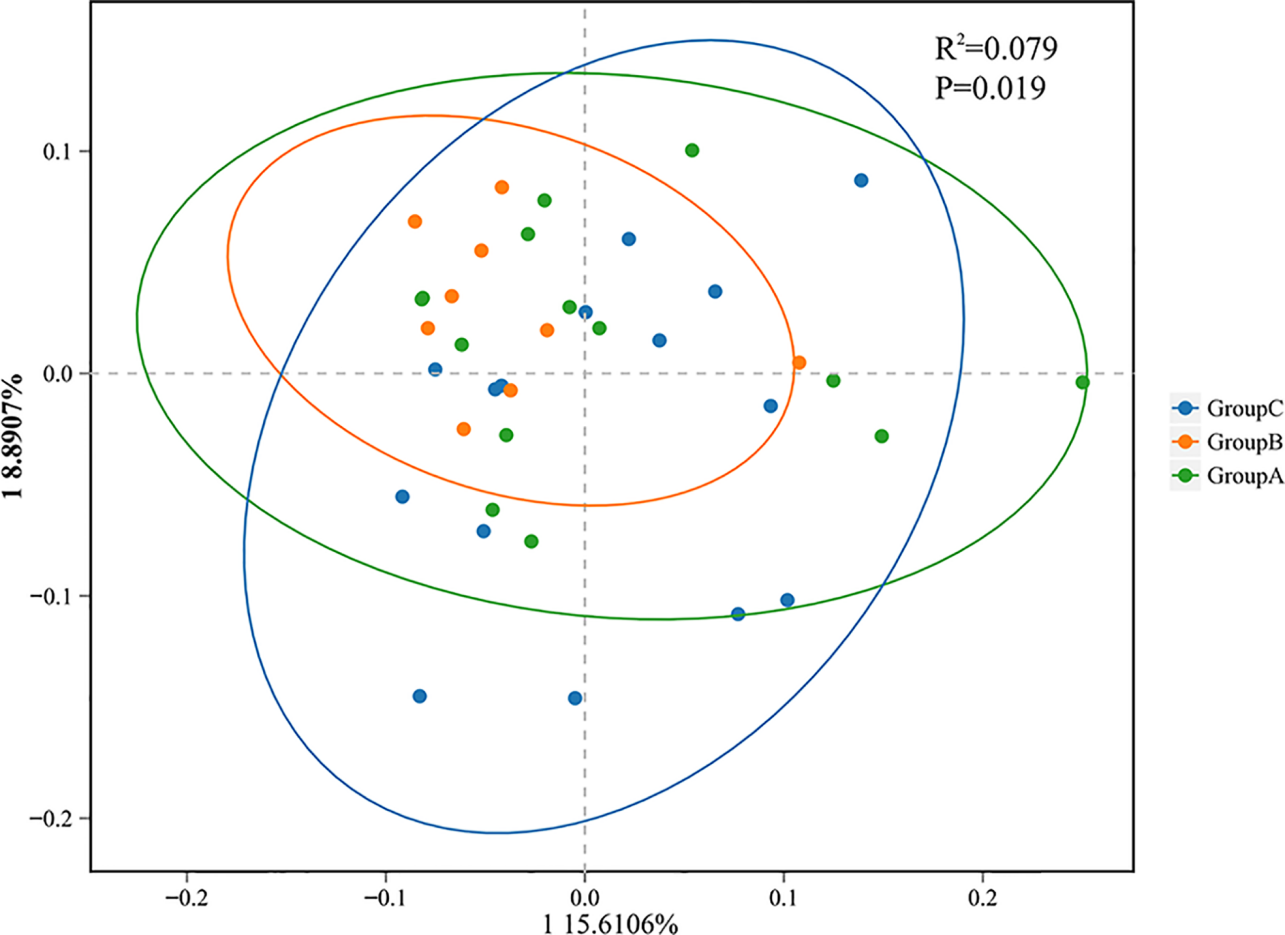
Principal coordinate analysis (PCoA) showing bacterial differences among groups. PCoA analysis is based on unweighted UniFrac distance matrices, and each symbol represents a sample. Blue rounds show samples from healthy controls (Group **C**), green rounds represent patients with thyroid cancer (Group **A**), and orange rounds represent patients with thyroid nodules (Group **B**). The healthy control group is separated from the others.

Beta-diversity analyses based on principal coordinates (PCoA) analysis suggested the differences in the composition and abundance of salivary microbiota of thyroid cancer, thyroid nodules, and healthy control groups. The differences between the three groups assessed by PERMANOVA based on unweighted UniFrac distance matrices were significant (p < 0.05, **Figure 4**). In a PCoA analysis, the closer the two samples were, the greater the similarity in community composition. The two circles for the thyroid cancer group and thyroid nodules group overlapped each other on the map, which indicated that the bacterial composition of the thyroid cancer group is similar to that of the thyroid nodules group. Moreover, the circle for the healthy control group was far from the other groups, suggesting that the saliva microbiota of the patients with thyroid cancer and thyroid nodules differed from healthy controls.

### Bacterial taxa differences in the salivary microbiota among the three groups

To further compare the structure of the salivary microbiota in the thyroid cancer group, thyroid nodules group, and healthy controls, we used a combination of linear discriminant effect size (LEfSe) and linear discriminant analysis discriminant effect size (LDA value of 2.0).

**Figure 5:**
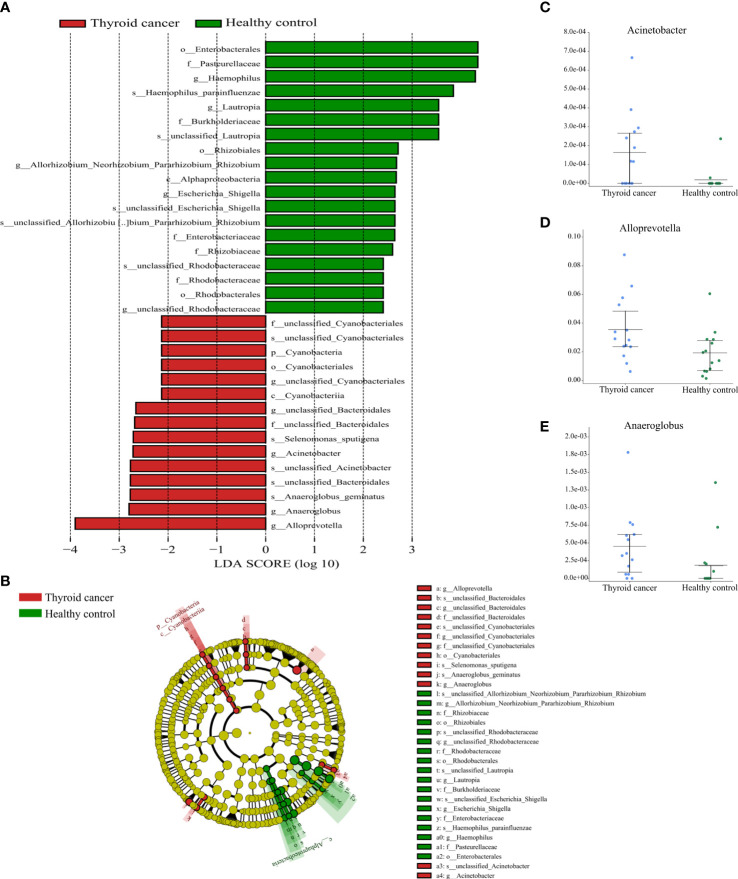
Partial bacterial taxa that differed significantly between healthy control and thyroid cancer groups. **(A)** Shows differentially abundant taxa in thyroid cancer and healthy control groups based on the linear discriminant analysis coupled with effect size (LEfSe). Taxa in this graph were both statistically significant (*P* < 0.05) and had LDA score above 2, which is considered a significant effect size. **(B)** A cladogram of the differential bacteria based on LEfSe. Circles from inside to outside represent species taxonomic levels from phylum to genera. The diameters of the circles represent the relative abundance. Red and green indicate enrichment in samples from the thyroid cancer patients and healthy controls, respectively. Yellow indicates no significant difference. **(C–E)** Relative abundance of taxonomy between thyroid cancer and healthy control groups was compared (*P* < 0.05).

**Figure 6:**
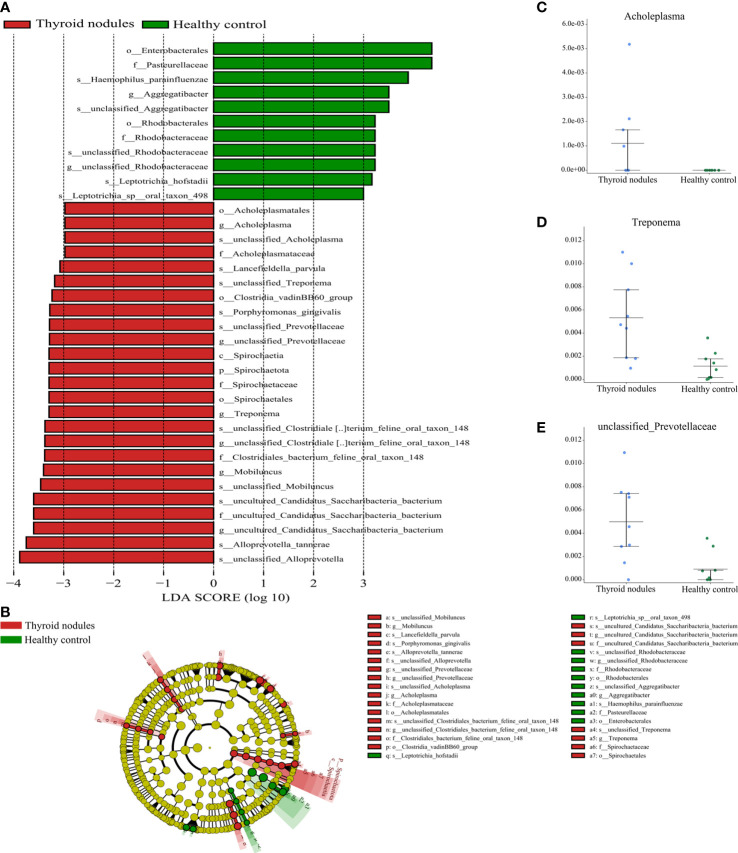
Partial bacterial taxa that differed significantly between healthy control and thyroid nodules groups. **(A)** Shows differentially abundant taxa in thyroid nodules and healthy control groups based on the linear discriminant analysis coupled with effect size (LEfSe). Taxa in this graph were both statistically significant (*P* < 0.05) and had LDA score above 2, which is considered a significant effect size. **(B)** A cladogram of the differential bacteria based on LEfSe. Circles from inside to outside represent species taxonomic levels from phylum to genera. The diameters of the circles represent the relative abundance. Red and green indicate enrichment in samples from the thyroid nodules patients and healthy controls, respectively. Yellow indicates no significant difference. **(C–E)** Relative abundance of taxonomy between thyroid nodules and healthy control groups was compared (*P* < 0.05).

In total, 34 taxa differed between the thyroid cancer and healthy controls; 15 taxa were significantly enriched in the thyroid cancer group (Kruskal-Wallis test, P < 0.05). One phylum Cyanobacteria, one class Cyanobacteria, one order (Cyanobacteriales), two families (unclassified Bacteroidales, unclassified Cyanobacteriales), and five genera (including Alloprevotella, Anaeroglobus, Acinetobacter, unclassified Bacteroidales, unclassified Cyanobacteriales) were significantly enriched in the thyroid cancer group compared with the healthy control group. In contrast, the microbiome of the healthy controls was mainly composed of the genera Haemophilus, Lautropia, Allorhizobium Neorhizobium Pararhizobium Rhizobium, Escherichia Shigella, and unclassified Rhodobacteraceae (**Figure 5**). Moreover, 36 taxa were siseven

gnificantly different between the thyroid nodules group and healthy controls (Kruskal-Wallis test, P < 0.05). The thyroid nodules group was dominated by genre uncultured Candidatus Saccharibacteria bacterium, unclassified Clostridiales bacterium feline oral taxon 148, Treponema, unclassified Prevotellaceae, Mobiluncus, and Acholeplasma, whereas the genera unclassified Rhodobacteraceae and Aggregatibacter dominated the healthy control group (**Figure 6**).

### Associations between clinical indices and salivary microbiome

**Figure 7:**
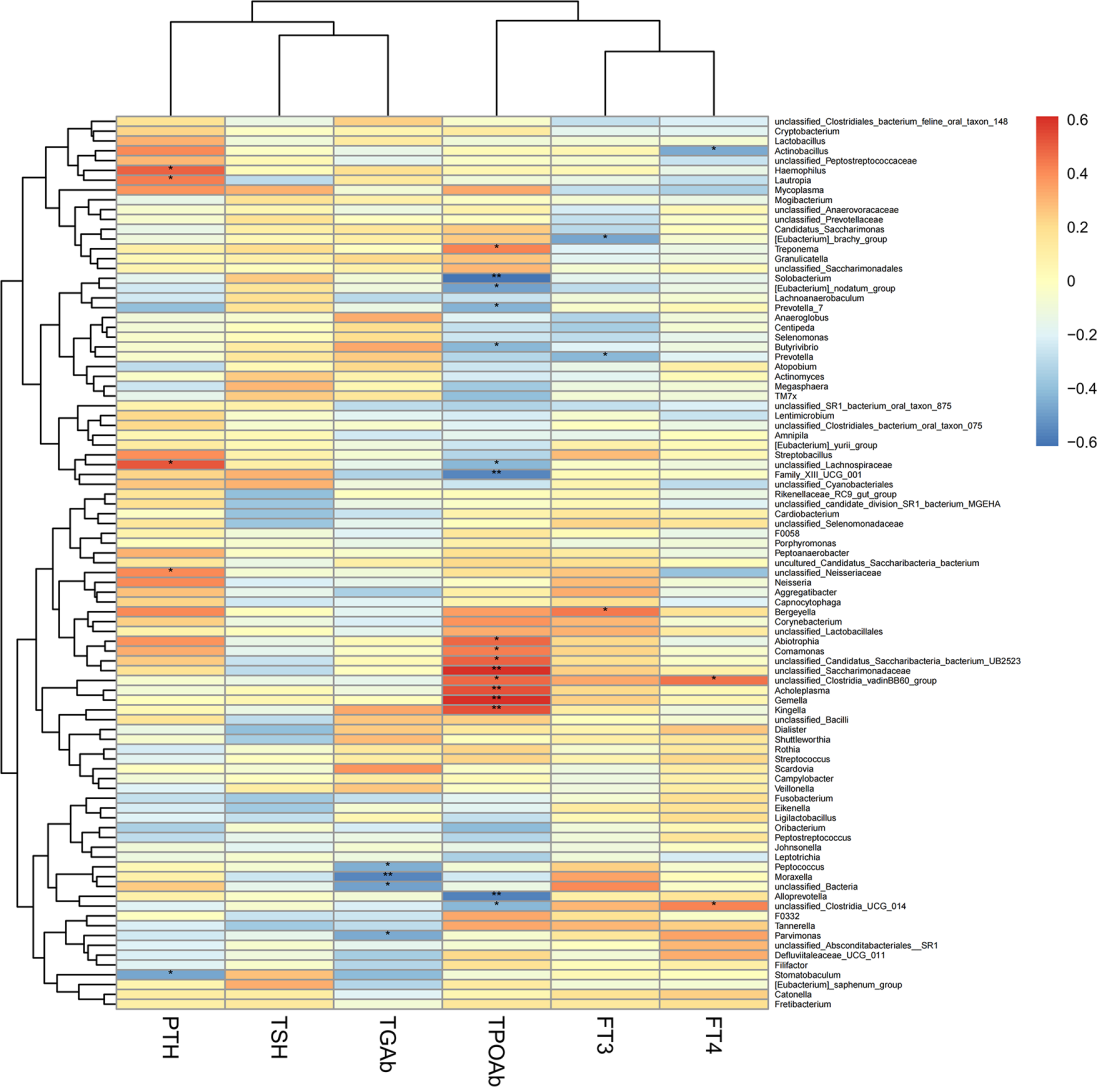
Heatmap of Pearson’s correlation analysis between the saliva microbiota at the genera levels of thyroid cancer and thyroid nodules and clinical variables. R value shows in different colors, red indicates positive correlation while blue indicates negative correlation. The darker the color, the greater the correlation coefficient. Species clustering trees were presented on the left side of the heat map. *P < 0.05; **P < 0.01.

**Figure 8:**
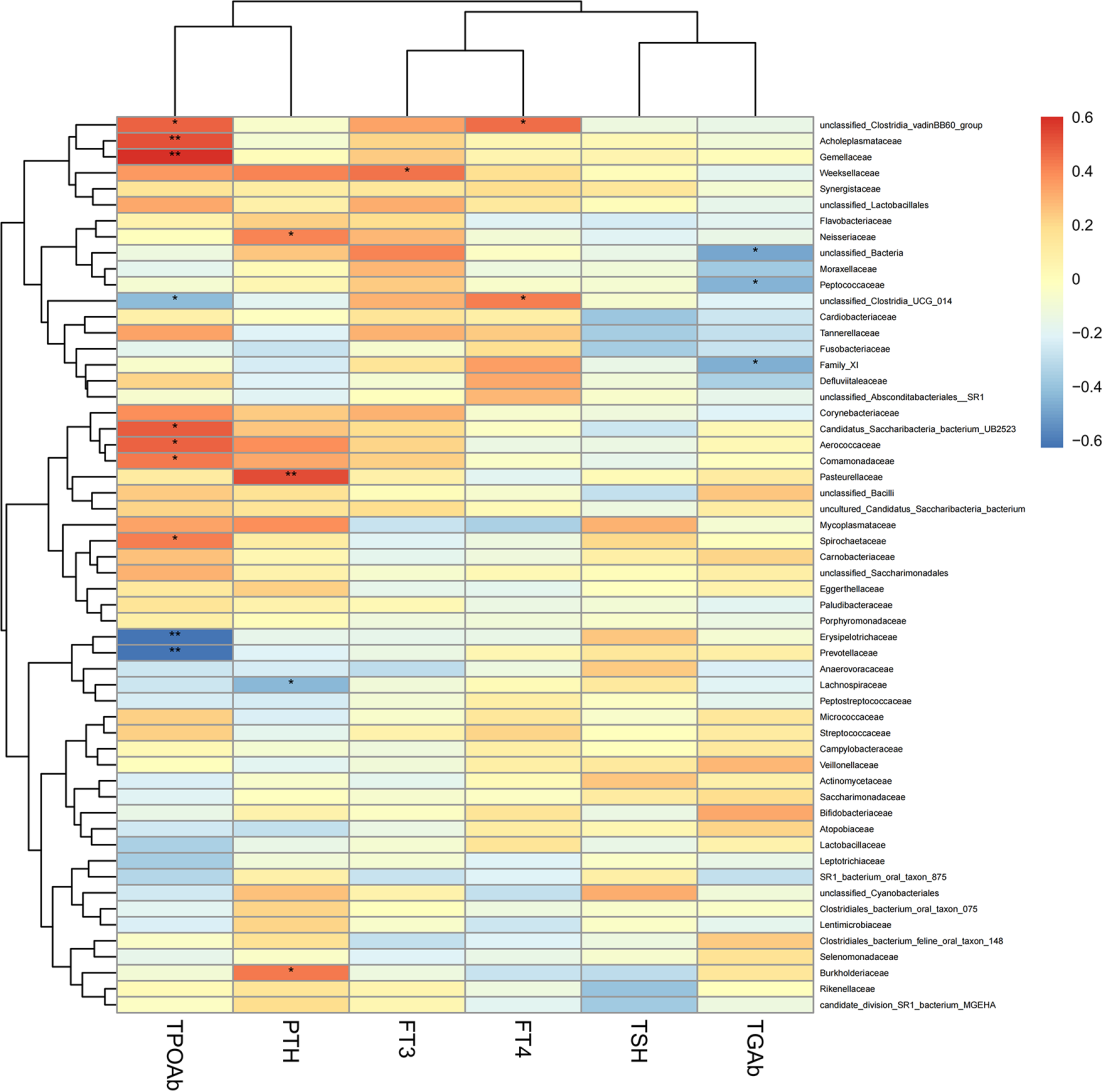
Heatmap of Pearson’s correlation analysis between the saliva microbiota at the family levels of thyroid cancer and thyroid nodules and clinical variables. R value shows in different colors, red indicates positive correlation while blue indicates negative correlation. The darker the color, the greater the correlation coefficient. Species clustering trees were presented on the left side of the heat map. *P < 0.05; **P < 0.01.

To explore the correlation between the relative abundance of saliva microbiota and clinical indices of patients with thyroid disease, we used Pearson’s correlation coefficient (p < 0.05, |correlation coefficient| > 0.4). The clinical parameters including TSH (thyroid-stimulating hormone), FT3 (Freetriiodothyronine), FT4 (Freetetraiodothyronine), TgAb (Thyroglobulin antibody), TPOAb (Thyroid peroxidase antibody), and PTH (Parathyroid hormone) and the genera with abundance ratios above 0.01 were visualized with a heatmap (**Figure 7**). TPOAb exhibited a positive correlation with the genera Treponema, Comamonas, Abiotrophia, unclassified Clostridia vadinBB60 group, unclassified Candidatus Saccharibacteria bacterium UB2523, Acholeplasma, Kingella, Gemella, and unclassified Saccharimonadaceae and showed a negative association with genera Solobacterium, Alloprevotella, Family XIII UCG 001, [Eubacterium] nodatum group, Prevotella 7, Butyrivibrio, unclassified Clostridia UCG 014, and unclassified Lachnospiraceae. The TGAb was negatively correlated with the genera Parvimonas, Peptococcus, and unclassified Bacteria. In addition, FT4 showed a positive connection with the genera unclassified Clostridia UCG 014 and unclassified Clostridia vadinBB60 group, but genera Actinobacillus was positively correlated with FT4 level. What’s more, a positive association can be observed between the genera Bergeyella and FT3. However, FT3 exhibited a negative correlation with genera Prevotella and [Eubacterium] brachy group. Additionally, the correlation between the genera Lautropia, unclassified Lachnospiraceae, and Stomatobaculum and the level of PTH was positive, but PTH was negatively correlated with unclassified Neisseriaceae and Haemophilus. At the same time, we observed that the genera Alloprevotella enriched in thyroid cancer was positively correlated with TPOAb levels. Similarly, the microbiota enriched in thyroid nodules, including genera Treponema and Acholeplasma, family Spirochaetaceae and Acholeplasmataceae, all exhibited a strong positive correlation with TPOAb (p < 0.05, **Figures 7**, **8**).

## Discussion

In this study, we characterized the richness and abundance of salivary microbiota of patients with thyroid cancer and thyroid nodules by 16S rRNA gene sequencing of saliva samples from patients with thyroid cancer and thyroid nodules, and healthy controls. The study demonstrated that the richness of saliva microbiota of the patients with thyroid cancer and thyroid nodules was higher than healthy controls, although the difference showed little significance. Several studies have investigated the relationship between the saliva microbiota and cancer (Galloway-Peña et al., 2017; Wang et al., 2019; Zhao et al., 2020). According to our data, the microbial richness was relatively higher in patients with thyroid cancer and thyroid nodules than in healthy controls, though there is no significant difference. Increased saliva microbiota richness has been documented in cancer cases and is believed to be one of the manifestations of oral microbial dysbiosis (Zhang et al., 2019; Wei et al., 2020). Notably, increasing evidence indicates a strong correlation between gut microbes and oral microbes (Arimatsu et al., 2014; Qin et al., 2014; Olsen and Yamazaki, 2019; Maki et al., 2020). There were some studies has reported the relationship between gut microbiota and patients with thyroid cancer and thyroid nodules, and some of our findings are consistent with the previous result. In 2018, Zhang et al. found an increase in the richness of the gut microbiota in patients with thyroid cancer and thyroid nodules by the Chao indices (Zhang et al., 2019). In the same year, another study indicated that patients with thyroid cancer show more extraordinary richness and diversity (α-diversity) of gut microbiota compared to healthy controls, as estimated by the Shannon and Chao induces (Feng et al., 2019). However, one opposite result was recently found in patients with thyroid cancer. Xia et al. found that the richness and diversity (α-diversity) of gut microbiota in thyroid cancer patients are significantly increased, as estimated by the Ace and Shannon index. The difference in the results may be related to the sampling area, time, and dietary habits of the patients. Still, most of the above results for the gut microbiota variation in thyroid cancer are consistent with our study.

In addition to changes in richness and diversity, we found a significant difference in the composition of salivary microbiota. The relative abundance of genera Alloprevotella, Anaeroglobus, Acinetobacter, unclassified Bacteroidales, and unclassified Cyanobacteriales was significantly higher in thyroid cancer patients. Genera Alloprevotella inhabits the human oral cavity and is related to but different from genera Prevotella (Downes et al., 2013). Previous studies evaluating the differences in saliva microbiota between patients with pancreatic adenocarcinoma (PDAC) and healthy people observed a significantly increased abundance of Alloprevotella among the patients with bloating in PDAC (Wei et al., 2020). Wu et al. found that the genera Alloprevotella was associated with a higher risk of cardia cancer (Wu et al., 2018). Zhang et al. reported that oral carcinoma showed a significantly higher abundance of genera Alloprevotella (Zhang et al., 2019). Additionally, a prior study indicated that the abundance of genera Alloprevotella as one of the periodontal pathogens has progressively increased along with the order of controls-PML (premalignant lesions)-OC-SCC (oral cavity squamous cell cancer) and contributed to enriched the variety of proinflammatory genes, which suggests a strong association between Alloprevotella spp. and cancer (Ganly et al., 2019). These studies suggest an increased abundance of Alloprevotella is associated with certain cancers. Additionally, recent studies indicate a strong relationship between the genera Alloprevotella and chromosomal aberrations, which have been shown to be related to the pathogenesis of anaplastic thyroid cancer and impact cancer incidence (Zitzelsberger et al., 2010; Saini et al., 2019; Druzhinin et al., 2020; Farkas et al., 2021). Based on these findings, we conclude that the increased abundance of Alloprevotella in the saliva is associated with thyroid cancer.

A study has demonstrated that the species Anaeroglobus geminatus are associated with the four lipid mediators in human periodontitis (Lee et al., 2021). To the best of our knowledge, inflammation is a hallmark of cancer, and the level of lipid mediators has a relationship with different cancers such as oral cancer, gastric cancer, melanomas, pancreatic cancer, colon cancer, liver cancer, and lung cancer (Sulciner et al., 2018; Biswas et al., 2022). Therefore, we hypothesized that variation in the species Anaeroglobus geminatus might have a relationship with cancer through lipid mediator levels.

Our study found the genera Acinetobacter was enriched in the saliva microbiota of patients with thyroid cancer. A 16s rRNA gene sequencing analysis of thyroid cancer tumor tissues and matched peritumor tissues reveals that the abundance of genus Acinetobacter is significantly enhanced in the tumor tissue. This result accords with our observation which suggested that the genera Acinetobacter could be strongly related to thyroid cancer patients. Besides, the genera Acinetobacter is confirmed to be the potential biomarkers of White-thin coating of GC (gastric cancer) patients using 16S and 18S rRNA high-throughput sequencing (Xu et al., 2019). A 16s rRNA gene sequencing analysis of saliva microbiota found that the genera Acinetobacter shows lower abundance in patients with lung cancer (Yang et al., 2018). The above studies indicate that the genus Acinetobacter plays a vital role in the carcinogenesis of different malignancies.

Regarding the changes in the salivary microbiota of patients with thyroid nodules, our study found that the abundance of Treponema and unclassified Prevotellaceae was significantly increased compared to the healthy population. In reviewing the literature, the genera Treponema shows potential pathogenic importance in autoimmune thyroid disease by molecular mimicry (Benvenga and Guarneri, 2016). Also, the prevalence and abundance of Treponema denticola evaluated by the real-time polymerase chain reaction of dental plaque in patients with esophageal cancer are higher than in healthy people (Kawasaki et al., 2021). The potential mechanisms for Treponema denticola contributing to carcinoma are associated with Treponema denticola chymotrypsin-like proteinase (Nieminen et al., 2018). Therefore, the virulence factor chymotrypsin-like proteinase of the Treponema genera enriched in thyroid nodules may affect the thyroid glands. Both unclassified Prevotellaceae and Prevotella are in the family prevotellaceae. Two studies indicate that the abundance of prevotellaceae is enriched in healthy people compared with thyroid cancer patients using a 16S rRNA analysis of the stool samples (Feng et al., 2019; Ishaq et al., 2022). In our study, we observed that the genera unclassified Prevotellaceae were enriched in salivary microbiota of thyroid nodules patients compared to healthy controls, which is somewhat different from the results of the two studies mentioned above. According to reports, the abundance levels of Prevotella are 11.56% in the microbiota of the throat, palatine tonsils, tongue dorsum, and saliva, and 3.16% in the fecal sample, which has the potential to explain the above opposite results (Segata et al., 2012). The reverse results may be related to the different effects of thyroid cancer and thyroid nodules on the body’s microbiota and, more likely, to the differences in the oral and gut microbiota. A previous study that evaluated the shifts of oral microbiota related to pregnancy has indicated that the abundance of genera Treponema spp and Prevotella spp is positively correlated with sex hormones (Lin et al., 2018). Moreover, some studies have suggested that estrogen might play a key role in thyroid nodules (Kim et al., 2010). The above studies show that the genera Treponema and unclassified Prevotellaceae may promote the progress of thyroid nodules.

In our study, we discovered that the genera Alloprevotella enriched in the thyroid cancer group showed a significant negative correlation with the level of TPOAb. Moreover, the genera Treponema (p < 0.05, r = 0.42) and genera Acholeplasma (p < 0.01, r = 0.53) enriched in thyroid nodules were positively correlated with TPOAb (p < 0.05). The family Spirochaetaceae (p < 0.05, r = 0.42) and Acholeplasmataceae (p < 0.01, r = 0.53) enriched in thyroid nodules patients were also significantly associated with the level of TPOAb. Hence, these results suggest that TPOAb is positively associated with parts of oral microbiota enriched in patients with thyroid nodules. However, current studies suggest that the relative role of TPOAb in thyroid nodules and thyroid cancer is unclear and remains to be further elucidated (Boi et al., 2017). Azizi et al. indicated that papillary thyroid carcinoma had a significant association with TSH levels and serum TgAb except for TPOAb in a prospective cytological study (Azizi et al., 2014).

In summary, our results indicated that the composition of saliva microbiota in patients with thyroid decrease is different from healthy controls, especially in some periodontal pathogenic bacteria. However, the mechanism is still not precise. While past studies have focused on changes in gut microbes in patients with thyroid cancer and thyroid nodules, our study presents the first characterization of salivary microbes in patients with thyroid cancer and thyroid nodules. However, the present study has several limitations that require further investigation. First, this study did not assess the function of bacterial and bacterial metabolites, and the mechanism is uncertain. Second, due to the limited conditions at the sampling time, we did not have a specialist to determine the patient’s periodontal condition, and the patient was unaware of any history of periodontal disease. Third, we included a relatively small sample size and did not validate across regions and time. Forth, the detection method is 16S gene sequencing. However, there are still many tentative names and unknown and unclassified bacteria in the database; hence further exploration of multiple groups such as macrogenes is recommended. Fifth, we were unable to observe changes in the microbiota after cancer or nodules development due to the experimental design. Last but not least, our findings should be validated with animal models.

## Conclusion

We identified the variation of salivary microbiota is connected with thyroid cancer and thyroid nodules. Based on our preliminary findings, the potential pathogens in thyroid cancer and thyroid nodules are active. However, it is still unknown whether thyroid disease causes dysbiosis of the oral microbiota or dysbiosis of the oral microbiota causes thyroid disease. These findings point the way forward for future research using modelled organisms to scrutinize the underlying mechanisms of the relationship between the salivary microbiome and thyroid disease.

## Data availability statement

The data presented in the study was deposited in NCBI Sequence Read Archive (SRA), accession number PRJNA863466.

## Ethics statement

The studies involving human participants were reviewed and approved by Medical Ethics Committee of Hospital of Stomatology, Jilin University. The patients/participants provided their written informed consent to participate in this study.

## Author contributions

NM, LZ, and JJ designed the study. JJ and YZ interpreted the data and wrote the paper. JJ gathered clinical data. JJ, YZ, DX, and QZ conducted analysis. All authors contributed to the article and approved the submitted version.

## Funding

This study was supported by Science and Technology Research Project of Jilin Provincial Education Department (JJKH20221095KJ).

## Conflict of interest

The authors declare that the research was conducted in the absence of any commercial or financial relationships that could be construed as a potential conflict of interest.

## Publisher’s note

All claims expressed in this article are solely those of the authors and do not necessarily represent those of their affiliated organizations, or those of the publisher, the editors and the reviewers. Any product that may be evaluated in this article, or claim that may be made by its manufacturer, is not guaranteed or endorsed by the publisher.
